# Rethinking strategies for solving thyroid dysfunction at the heart of cardiovascular disease

**DOI:** 10.1186/s10020-025-01351-x

**Published:** 2025-12-02

**Authors:** Viswanathan Rajagopalan, Kaie Ojamaa, A. Martin Gerdes

**Affiliations:** 1 New York Institute of Technology College of Osteopathic Medicine at A- State, Jonesboro, AR United States of America; 2 New York Institute of Technology College of Osteopathic Medicine, Old Westbury, NY United States of America

**Keywords:** Thyroid hormones, Heart failure, Acute coronary syndromes, T3, Triiodothyronine, T4, Thyroxine, Biomarkers, BNP

## Abstract

Throughout a person’s lifetime, thyroid hormones (THs) have an outsized impact on cardiovascular health from prenatal heart development to adult cardiac contractile function and blood pressure regulation. Maintaining a healthy functioning hypothalamic-thyroid axis is crucial for preventing cardiac-related and all-cause mortality. Patients with moderate to severe heart failure (HF) often manifest with low or borderline-low TH function. In this review article, we examine the potential of TH therapy in HF management by highlighting outcomes from recent clinical studies. We also address the need for a serum-based biomarker such as brain natriuretic peptide (BNP) that indicates disease stage of HF and that also correlates with cardiac tissue TH status. Recent and newer therapeutic strategies (including the combination of Triiodothyronine and Thyroxine) to advance the management of patients living with HF are proposed including a reassessment of what is normal thyroid status in these patients and the potential of TH treatment.

## Highlights

Significant clinical evidence indicates adverse cardiovascular outcomes from low TH function even within reference ranges.

Heart-derived serum biomarker BNP inversely correlates with serum T3 and T3/T4 ratio in failing hearts.

Better bioavailable formulations and combinations of THs show promise in treating CV diseases.

Some of the most profound manifestations of thyroid dysfunction are observed on cardiac hemodynamics and vascular reactivity. Thyroid hormones (THs) have been used clinically for the treatment of primary overt hypothyroidism (low serum TH levels) for a long time; however, neither THs nor their analogs have made it to mainstream clinical application for the management of cardiovascular (CV) disorders without any prior primary thyroidal illness. This review provides a brief overview of published clinical studies, and explores opportunities to utilize TH treatments for CV diseases.

## A brief primer on CV disorders and impaired thyroid function

The most common form of thyroid dysfunction is hypothyroidism and this prevalence increases with age. Depending on the definition used and the population studied, the prevalence of hypothyroidism (subclinical and overt) may vary from 0.3 to 3.7% in the United States and 0.2–5.3% in Europe (Taylor et al. 2024; Canaris et al. 2000). The prevalence of hypothyroidism can be up to 17% in females and 5.9% in males, and the occurrence of subclinical hypothyroidism (SCH) is about 4–20% of the US population (Wyne et al. 2022). In addition, SCH may progress to overt hypothyroidism in about 2–5% of cases and is more likely to occur in patients with thyroid peroxidase antibodies (Chaker et al. 2022; Vanderpump et al. 1995; Khandelwal And Tandon 2012). Low triiodothyronine (T3) syndrome (LT3S) presents with reduced serum total T3, and with thyroid stimulating hormone (TSH; thyrotropin) and free thyroxine (fT4) within the normal reference ranges (Kannan et al. 2018). Overt hypothyroidism presents with increased TSH and decreased THs (fT4), whereas SCH presents with increased TSH, but with fT4 in the normal range (Chaker et al. 2022). A TSH reference range of 0.4–4.0 mIU/L and an fT4 reference range of 0.76–1.46 ng/dL are generally accepted (Taylor et al. 2024; Garber et al. 2012).

## Molecular actions of thyroid hormones

The thyroid gland produces and secretes thyroxine (T4) as the principal hormone and releases it into the bloodstream (Braverman 2020). T4 production in the gland is regulated by thyroid stimulating hormone (TSH; thyrotropin) that is derived from the brain pituitary gland. The biologically active form of THs is triiodothyronine (T3), which is predominantly produced in peripheral tissues from the conversion of T4 to T3 by deiodinase enzymes. Cardiomyocytes possess the necessary machinery to sense, process, and respond to signals from THs (Gerdes and Iervasi 2010; Gerdes and Ojamaa 2016; Rajagopalan and Gerdes 2020). Briefly, T3 actions are mediated by specific receptors, TRα and TRβ, each with distinct isoforms that are localized to the cell nucleus where they bind to and regulate a unique set of genes (Dore And Mittag 2024; Ojamaa And Carrillo-Sepulveda 2020). Multiple studies have shown that T3 regulates key signature cardiomyocyte genes including myosin heavy chains (MHC), sarcoendoplasmic reticulum calcium adenosine triphosphatase (SERCA2), sodium/potassium adenosine triphosphatase, troponins, sodium/calcium exchanger (NCX), beta-1 adrenergic receptor (β1-AR), Matrix Metalloproteinase 2 (MMP), Tissue Inhibitor of Metalloproteinase 1–4 (TIMP), Phospholamban, voltage-gated potassium channels Kv1.5, Kv4.2, Kv4.3. Expression of these genes may be impaired in the diseased heart (Ojamaa And Carrillo-Sepulveda 2020; Schwartz And Mercadier 1996; Yamakawa et al. 2021; Jabbar et al. 2017). In addition, non-genomic mechanisms are involved in mediating T3 effects on the heart including alterations in the organization of Ca^2+^ ion channels at dyad structures and their co-localization with other Ca^2+^, Na^+^ and K^+^ion channels and activities of ion currents (Yamakawa et al. 2021; Gilani et al. 2021; Charest et al. 2024; Janssen et al. 2017). Thyrotropin (TSH) acting through its receptor in the heart has been shown to modulate the activities of repolarizing currents such as Ito and IKs (Casis et al. 2024; Alonso et al. 2015; Fernandez-Ruocco et al. 2019). Data from numerous studies support cellular mechanisms by which THs regulate cardiac hypertrophy by signaling through p85α, FoxO1–deiodinase 2, angiotensin-1 receptor, and extracellular signal-regulated kinase pathways (Ferdous et al. 2020; Kenessey and Ojamaa 2006; Diniz et al. 2009; Pantos et al. 2007; Ojamaa 2010; Rajagopalan and Gerdes 2015). Furthermore, THs have been shown to improve myocardial bioenergetics and mitochondrial function (Rajagopalan and Gerdes 2015; Marin-Garcia 2010; Madathil et al. 2015; Tan et al. 2019). In addition, THs are involved in the regulation of microvascular blood flow, vascular lipids, cardiac metabolism, and collagen content (Janssen et al. 2017; Ferdous et al. 2020; Rajagopalan and Gerdes 2015; Gerdes 2025; Traub-Weidinger et al. 2012; Kotwal 2020; Zhou et al. 2023; Russo 2021; Wu et al. 2007). Differentiation and maturation of cardiomyocytes derived from stem cells and maturation of neonatal cardiac myocytes have an absolute requirement for T3 activity (Rajagopalan and Gerdes 2015; Gerdes 2025; Yang et al. 2014; Parikh et al. 2017; Lee et al. 2010; Ulivieri et al. 2022; Jackman et al. 2018).

Many of the phenotypic and functional changes observed in the failing heart are similar to those seen in hypothyroidism (Gerdes and Iervasi 2010; Gerdes and Ojamaa 2016; Schwartz And Mercadier 1996; Yamakawa et al. 2021). Several studies have established that HF is characterized by the phenomenon of re-expression or reactivation of fetal genes, including beta-MHC, ANP, BNP, miRNAs, α-skeletal actin, hyperpolarization-activated cyclic nucleotide-gated channel, T-type Ca^2+^ channel, and suppression of adult cardiac genes such as sarco-endoplasmic reticulum Ca2 + ATPase, alpha MHC (Gerdes and Iervasi 2010; Janssen et al. 2017; Wu et al. 2007; Weltman et al. 2013, 2014; Mantzouratou et al. 2020; Pol et al. 2011). We have shown that hypothyroidism leads to severe, progressive systolic dysfunction and increased chamber diameter/wall thickness ratio in spite of a reduction in cardiac mass. The chamber dilatation in the hypothyroid condition in animal models is due to myocyte elongation which is characteristically typical of HF (Tang et al. 2005). Taken altogether, these compelling data support a central regulatory role of THs on cardiac structure and function in the adult, and on the maturation of the developing heart. Thus, normal thyroid function is paramount in maintaining cardiac health.

## Clinical presentation of thyroid dysfunction

Hypothyroidism presents with reductions in ejection fraction (EF) and heart rate, diastolic hypertension, prolonged QT interval, attenuated precordial activity, cold intolerance, fatigue, slowing of metabolic processes, and dyslipidemia. In contrast, hemodynamic changes in hyperthyroidism, resulting from excess THs, are generally opposite to those of hypothyroidism and are often accompanied by more pronounced symptoms (based on the age and severity of hyperthyroidism) (Gerdes and Ojamaa 2016; Rajagopalan and Gerdes 2015; Pingitore et al. 2023; Klein and Ojamaa 2001). In a meta-analysis of 37 cohort studies, the pooled hazard ratio for overt hyperthyroidism compared with the control group was 1.11 (95% confidence interval [CI], 1.03 to 1.19) for ischemic heart disease, 1.35 (95% CI, 1.03 to 1.75) for stroke, and 1.20 (95% CI, 1.00 to 1.46) for CV mortality (Sohn et al. 2020). For subclinical hyperthyroidism, the pooled hazard ratio was 1.24 (95% CI, 1.07 to 1.45) for ischemic heart disease.

Observational studies have shown that reduction in T3 was proportional to the severity of left ventricular (LV) dysfunction in both HF with reduced EF (HFrEF) and preserved EF (HFpEF) (Pingitore et al. 2006; Chen et al. 2015; Selvaraj et al. 2012; Turic et al. 2023). Although the reductions in circulating T3 levels were associated with significant increases in the BNP levels in both HFrEF and HFpEF, both these forms of HF present with distinct pathophysiology (Pingitore et al. 2006; Chen et al. 2015; Selvaraj et al. 2012; Turic et al. 2023). For instance, HFpEF patients with lower T3 more frequently had significant diabetes mellitus. In addition, they were older and more symptomatic, and severe diastolic dysfunction, higher mitral E velocity, shorter deceleration time, and higher pulse pressure/stroke volume ratio were all associated with lower T3 levels. We previously showed that THs regulate microvascular blood flow (Weltman et al. 2014; Khalife et al. 2005). More research investigating the roles of low cardiac tissue T3 levels (discussed later) in impaired cardiac microvascular blood flow in CV diseases is needed. This will help better distinguish between both forms of HF, and also in diabetic cardiomyopathy, where microvascular blood flow impairment may play a major role (Del Buono et al. 2021; Kibel et al. 2017). Furthermore, a recent published report from our group indicates that long noncoding RNA mechanisms downstream of the TH signaling pathway may play important roles in distinguishing the pathophysiology of HFrEF versus HFpEF (Chakraborty et al. 2025).

A meta-analysis (Ning et al. 2017) with a total of 1,898,314 subjects from 55 studies showed that overt hypothyroidism is associated with an increased risk of cardiac mortality (relative risk [RR]: 1.96), all-cause mortality (RR: 1.25), myocardial infarction (MI; RR: 1.15), and ischemic heart disease (RR: 1.13). In addition, cardiac patients with hypothyroidism experienced greater risks of cardiac mortality (RR: 2.22) and all-cause mortality (RR: 1.51). SCH, especially with TSH level ≥ 10 mIU/L) was also associated with increased risks of ischemic heart disease and cardiac mortality. A study (Evron et al. 2022) of 705,307 US veterans showed increased risks of CV mortality in both overt hypothyroidism (TSH > 5.5 mIU/L or fT4 < 0.7 ng/dL) and overt hyperthyroidism (TSH < 0.5 mIU/L or fT4 > 1.9 ng/dL). In a study of patients with New York Heart Association-class III/IV HF and a subgroup with type-2 diabetes mellitus (DM), the authors reported that decreased free T3 was associated with an increased rate of short-term adverse outcomes over a 6-month follow-up period. This indicates the coexistence of thyroid dysfunction and poor CV outcome (Chen et al. 2015). In a meta-analysis involving 11,753 euthyroid patients and 1,132 SCH patients undergoing percutaneous coronary intervention, SCH was associated with increased risks of CV mortality, all-cause mortality, and repeat revascularization compared to euthyroid patients (Ang et al. 2023). Another meta-analysis of non-cardiac patients with SCH showed significantly impaired systolic and diastolic function (Li et al. 2023). In patients with acute decompensated HF, SCH (Hayashi et al. 2016) and hypothyroidism were independent predictors of adverse CV outcomes and all-cause mortality, respectively. Atrial fibrillation (AF) has been reported in 2.5% of patients with overt hypothyroidism, 5.3–6.5% with SCH, 4.6–13.8% with overt hyperthyroidism, and 4.2–12.7% with subclinical hyperthyroidism (Selmer et al. 2012; Auer et al. 2001; Bekiaridou et al. 2022; Singh et al. 2024; Kostopoulos et al. 2024; Baumgartner et al. 2017). Among new-onset arrhythmias associated with thyroid dysfunction, atrioventricular block (1.49%) is the most frequent followed by AF (0.92%) (Doshi et al. 2020). Low T3 Syndrome may be secondary to peripheral dysregulation of the conversion of T4 to T3 and could occur in up to 30% of CV patients, including heart failure (HF) (Kannan et al. 2018; Iervasi et al. 2003). LT3S has been reported to be prevalent among patients with hypertrophic cardiomyopathy and independently associated with an increased risk of sudden cardiac death (SCD) events and worsening HF. A single-center retrospective study evaluating thyroid dysfunction on disease prognosis of 3733 patients with pre-existing HF requiring hospitalization, found that LT3S, SCH, and hypothyroidism were independently associated with poor outcomes (Zhou et al. 2023).

Langen et al. (Langen et al. 2018) reported that high TSH at baseline was also associated with a greater risk of SCD (Hazard Ratio: 2.28) compared with TSH within the reference range. Furthermore, Brugada syndrome (Theisen et al. 2023) is an inherited disorder that can cause ventricular fibrillation and SCD in individuals with otherwise macro-structurally normal hearts. Importantly, four case studies (Kitahara et al. 2008; Zhao et al. 2012; Taira et al. 2010; Bioletto et al. 2024) independently reported that Brugada-electrocardiographic waveforms disappeared with the normalization of thyroid function in 33-, 46-, 52-, and 77-year-old males presenting with hypothyroidism. The reversal of electrocardiographic modifications was documented even at a low subtherapeutic dose. This suggests that hypothyroidism may be involved in Brugada mechanisms and presentations, which may predispose patients to SCD via potential electrophysiological alterations. More studies are needed to understand the role of THs in SCD.

## The case for TH therapy in CV disorders

Several preclinical studies have reported reduced cardiac tissue T3 and T4 levels in disease models including diabetic cardiomyopathy, MI, ischemia-reperfusion injury, hypertension and hypothyroidism, and these are discussed in multiple reviews (Rajagopalan and Gerdes 2020; Gerdes 2014; Gerdes et al. 2021). This tissue level hypothyroidism is attributed to an increase in type 3 deiodinase in the myocardium that inactivates bioactive T3 and T4 (Russo et al. 2021; Weltman et al. 2014; Salvatore et al. 2022; Janssen et al. 2013, 2016). Rats subjected to experimental anti-thyroid drug exposure were shown to have low cardiac tissue T3 levels and depressed cardiovascular physiological markers (Weltman et al. 2013). Importantly, T3 supplementation restored these and other cardiac functional deficits. Along with low tissue T3 and activated type 3 deiodinase, similar alterations and subsequent restoration with treatment were reported in a model of diabetic cardiomyopathy and in HF after MI (Weltman et al. 2014; Pol et al. 2011). Furthermore, T3 treatment was shown to attenuate maladaptive changes in the myocardial T-Tubule/Sarcoplasmic reticulum (TT/SR) ultrastructure in MI-induced HF in rats (An et al. 2019). Low cardiac tissue T3 has been demonstrated in chronic adrenergic stimulation-induced cardiac hypertrophy (Simonides et al. 2023). High-resolution single molecule localization microscopy showed that T3 treatment of hypothyroid rats normalized T-Tubule periodicity, improved cardiomyocyte contractility and excitation-contraction (EC)-coupling by colocalizing L-type Ca^2+^channels and junctophilin-2 (Jph) with ryanodine receptor (RyR) clusters at the TT-SR dyad structures (Gilani et al. 2021; Charest et al. 2024). Additionally, noncoding RNAs including novel long noncoding RNAs and circRNAs, and microRNAs, have been shown to be regulated by T3 indicating another regulatory mechanism of action (Rajagopalan et al. 2023). At least two clinical studies have assessed T3 levels in human heart tissues from patients with HF and have reported reduced T3 content (Gil-Cayuela et al. 2017, 2018). Other plausible mechanisms may involve the effect of heart on brain function in CV disease, causing altered hypothalamic-pituitary activity. Low serum T3, and/or reduced tissue uptake would also diminish T3 effects on cardiac function. Much has still to be learned about the heart-brain connection.

Multiple clinical studies have suggested that Levothyroxine (L-T4) therapy is cardioprotective in both overt hypothyroidism and SCH patients including those with dyslipidemia and impaired coronary microvascular dysfunction (Traub-Weidinger et al. 2012; Kotwal et al. 2020; Yazici et al. 2004; Wang et al. 2024, 2022; Monzani et al. 2001). L-T4 improved serum lipid profile, myocardial lipid load, and cardiac output in the hypothyroid patients. This opens up the possibility of treating conditions with microvascular impairment including diabetes mellitus and HFpEF (Kotwal et al. 2020; Zhou et al. 2023; Selvaraj et al. 2012; Gerdes 2014; Gerdes et al. 2021). However, treatment with inappropriate dosing of L-T4 has been associated with palpitations, accelerated heart rhythm, atrial fibrillation, HF, and increased CV morbidity and mortality (Feldt-Rasmussen et al. 2024; Du et al. 2022; Nanjappa And Rodrigues 2024). The Thyroxine in Acute Myocardial Infarction (ThyrAMI) study (Razvi et al. 2020) (1806 patients) revealed that the timing of sample blood collection significantly impacted the diagnosis of SCH in acute MI. The risk of diagnosing SCH was reported to be highest when measured between 00:01 h and 06:00 h and lowest between 12:01 h and 18:00 h. Therefore, it is possible that studies that have not followed specific sample collection times may have reported varied outcomes in assessing the safety and efficacy of TH treatment in CV disorders. Importantly, subgroup analyses based on patient background, disease severity, drug formulation, and other variables may help reveal the best indications for therapy in specific patient subpopulations.

Guidelines from the American Thyroid Association (ATA), the European Thyroid Association (ETA), and the National Institute for Health and Care Excellence (NICE) recommend that hypothyroidism be treated with L-T4. In hypothyroid patients with coexistent coronary artery disease, the guidelines recommend starting low-dose L-T4 and increasing the dose slowly. Additional measures to treat CV disorders are indicated if a full dose is not tolerated. For SCH, L-T4 can be considered at TSH levels of 10 mIU/L or higher measured on two separate occasions. For SCH with coexistent CV disorders, the guidelines recommend treating (i) all patients, (ii) or consider treating those with TSH levels of 4.5–10 mIU/L with atherosclerotic CV disease, HF, or associated risk factors for these conditions, and (iii) treating patients with TSH levels of 4.5–10 mIU/L who are < 65 years of age with increased CV risk (e.g. previous CV disease, DM, dyslipidemia, hypertension, metabolic syndrome), particularly with TSH level persistently > 7 mIU/L (Taylor et al. 2024; Garber et al. 2012, 2012; Cappola et al. 2019; Brenta et al. 2013; Jonklaas et al. 2014). However, treatment with L-T4 alone may be sub-optimal for a substantial number of patients due to possible insufficient conversion of T4 to T3 in target organ systems such as the heart (Rajagopalan and Gerdes 2020; Gerdes 2014; Salvatore et al. 2022; Paolino et al. 2017).

Reductions in serum T3/T4 ratio suggest poor peripheral thyroid conversion and worse outcomes (Rajagopalan and Gerdes 2020; Gerdes 2014; Salvatore et al. 2022). Accelerated TH inactivation was reported in the myocardium of aortic stenosis patients, likely due to increased type-3 deiodinase that converts T4 to reverse (r)T3, an inactive hormone (Paolino et al. 2017). These patients exhibited increased serum rT3 and decreased serum T3/rT3 ratio, suggesting impaired conversion to bioactive T3. Studies indicate worse HF outcomes in the higher quartile or tertile of the TSH reference range (Chen et al. 2014). Other mechanisms of TH protection include reductions in circulating catecholamines, aldosterone, and lipids, and improvements in arteriolar microcirculation and metabolism (Zhou et al. 2023; Pingitore et al. 2008; Hamilton et al. 1998; Biondi et al. 2023). L-T4 treatment alone may not be sufficiently efficacious in all patients with CV disease since it has been observed that T4-treated patients with hypothyroidism have relatively lower serum T3 than the general population, thus suggesting that either T3 therapy alone, or combined T4 plus T3 therapy (discussed later) may be better (Salvatore et al. 2022).

Multiple clinical studies have shown the beneficial effects of T3 therapy in CV patients. T3 administration has been shown to be safe, well-tolerated and beneficial in patients with dilated cardiomyopathy, stable and/or advanced HF (Pingitore et al. 2008; Hamilton et al. 1998; Novitzky et al. 1989; Amin et al. 2015). Notably, the THIRST study (Pingitore et al. 2019) (Thyroid Hormone Replacement Therapy in ST Elevation Myocardial Infarction) showed that oral L-T3 (liothyronine; maximum 15 mcg/m^2^/day; 6 months) safely improved regional cardiac dysfunction including wall motion score index and stroke volume in patients with acute ST-elevation MI and LT3S. Tharmapoopathy et al. (Tharmapoopathy et al. 2022) conducted a systematic review and meta-analysis investigating 12 randomized clinical trials of high to moderate-quality evidence in 1093 adults undergoing cardiac surgery with T3 treatment. T3 improved cardiac index without causing detrimental effects on heart rate, risk of in-hospital AF or mortality. The TRICC-2 trial of T3 Supplementation in Infants and Children Undergoing Cardiopulmonary Bypass showed various benefits on outcomes and demonstrated that T3 supplementation was safe, even using dosing strategies that increased T3 above baseline (Portman et al. 2023). Taken together, T3 supplementation has been validated to be safe (no significant differences in arrhythmias or other sentinel adverse events) among diverse adult and pediatric populations with chronic/critical illnesses, particularly in patients with HF (Portman et al. 2023; Radman and Portman 2016; Klemperer et al. 1995, 1996; Chowdhury et al. 2001). Importantly, a long-term Scotland-based observational study (Leese et al. 2016) compared patients consuming L-T4 only (*n* = 33,955) vs. L-T3 only (*n* = 73) or combined L-T4 + L-T3 (*n* = 327) with a maximum follow-up of 17.3 years and a mean follow-up of 9.3 years (SD 5.6). The objective of the study was to investigate adverse outcomes in patients receiving L-T3 compared to those (in large numbers) receiving only L-T4. They reported that patients taking L-T3 (with or without L-T4), did not reveal higher mortality or morbidity risk associated with CV disease, AF, DM, or fractures after adjustment for age and other baseline confounding variables, compared to patients taking only L-T4. Taken together, these indicate that T3 may be tolerable and not deleterious for CV health.

## Serum biomarkers to assess myocardial TH function and to monitor T3 therapy

One of the challenges in assessing TH treatment effects on CV function in disease is that hearts can suffer from low tissue TH concentrations even though serum THs may be normal (Gerdes 2014; Gerdes et al. 2021; Liu et al. 2008). The difficulty in predicting when and to what extent cardiac T3 is reduced, without a tissue biopsy, necessitates serum biomarkers that can correspondingly reflect the tissue T3 levels. The ideal serum biomarker would be one secreted by the heart in response to low tissue T3 content, and subsequently, its release from the heart be diminished by restoration of THs following T3/T4 treatment.

B-type natriuretic peptide (BNP) is a hormone synthesized and secreted by the heart in response to pressure or volume overload, and in response to inflammation as occurs in HF. Large Danish and US-based studies indicated that BNP is a predictor and independent risk factor for all-cause and CV disease mortality, and could improve risk stratification of pre-HF patients (Hejl et al. 2018; Jia et al. 2023).

Clinically, a significant negative correlation between T3 and BNP has been reported in idiopathic LV dysfunction (Pingitore et al. 2006; Amin et al. 2015). In the aforementioned randomized, placebo-controlled study (Pingitore et al. 2008) of patients with chronic HF with LT3S, L-T3 infusion significantly decreased plasma NT-proBNP, noradrenaline, and aldosterone. Turić et al. (Turic et al. 2023) and Selvaraj et al. (Selvaraj et al. 2012) also reported that serum total T3 was inversely associated with NT-proBNP in HF with reduced or preserved EF. In a study (Chen et al. 2015) investigating chronic HF with type-2 DM, NT-proBNP was inversely associated with both LV EF and free T3, and correlated with New York Heart Association disease class. Thus, BNP which is widely measured in clinical laboratories could be used to infer cardiac tissue TH status. Non-thyroidal illness with low free T3 was detected in a significant proportion of children with congenital heart disease and congestive HF. In addition, free T3 levels were significantly associated with NT-BNP levels and thus the severity of HF (Sahoo et al. 2024). Although BNP may be altered in other conditions such as volume status, tachycardia, inflammation, and renal dysfunction (Nishikimi And Nakagawa 2021), the combination of low T3 with high BNP could indicate worse long-term outcomes. Recently we addressed the mechanisms linking THs and BNP expression in preclinical models of heart disease (Wang et al. 2020). The data showed that T3 treatment reduced cardiac tissue BNP expression and decreased serum BNP levels concomitantly with improved cardiac function. This inverse relationship between T3 and BNP opens the possibility that BNP could be a reliable serum biomarker to safely and precisely titrate the T3 dose in the treatment of patients with advanced HF diagnosed with SCH or LT3S.

## What is the way forward?

In light of the present discussion, both experimental and clinical evidence support taking into consideration an assessment of thyroid function in the setting of a diagnosis of HF. HF may be caused by various diseases affecting the myocardium, involving heart valves and vessels, or by metabolic disorders. Thyroid dysfunction may co-exist as either causal or consequence of heart disease, and its function can be easily measured by standard laboratory tests. Results indicating LT3S, SCH, or T3 in the lower quartile range should be considered in the management of the disease.

**Fig. 1:**
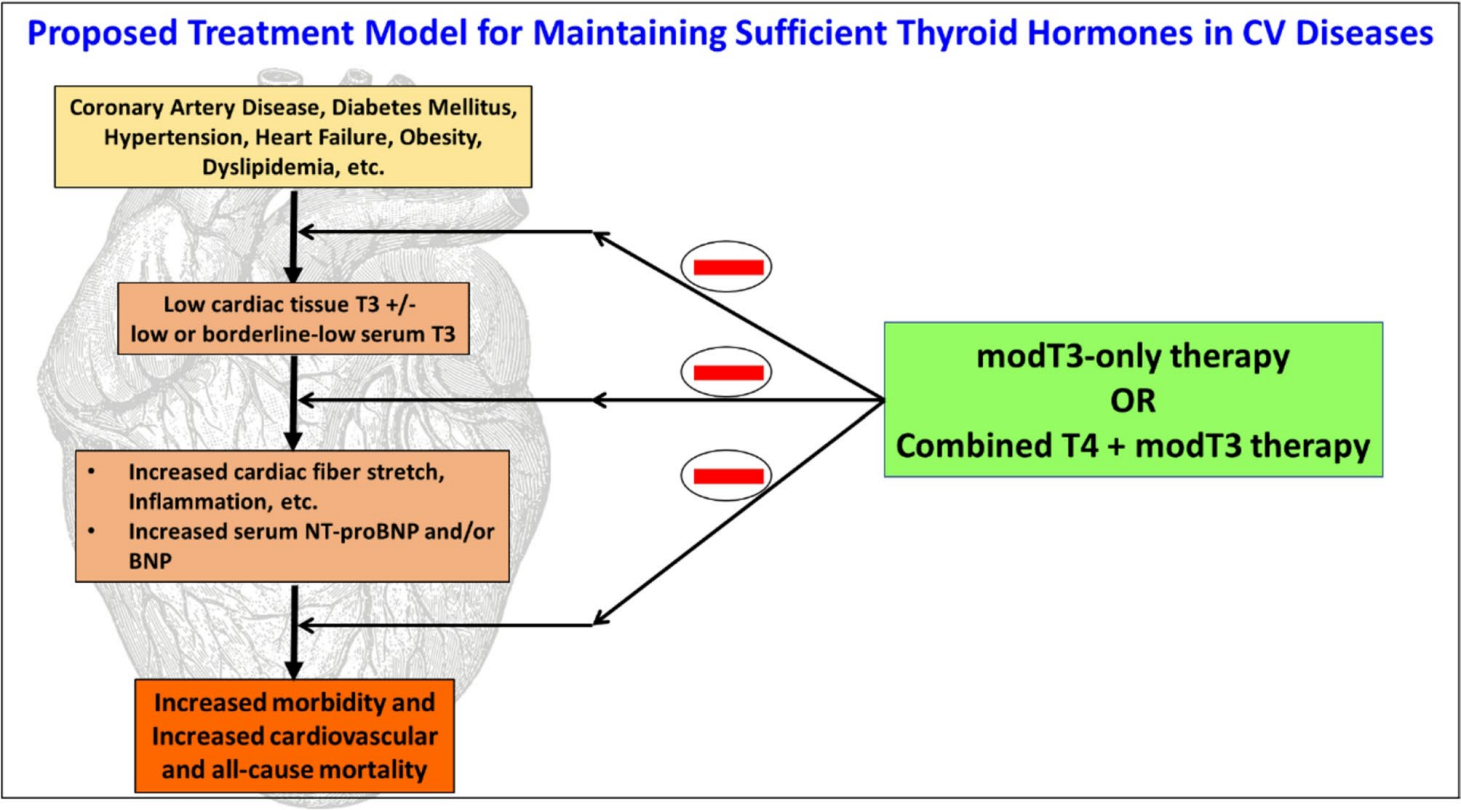
Proposed model for sufficient thyroid hormones in cardiovascular diseases: The importance and mechanisms of thyroid hormones in cardiovascular diseases, and strategies to monitor and restore its balance are presented; modT3 – modified Triiodothyronine; T4 – Thyroxine; BNP – Brain Natriuretic Peptide; LV – Left Ventricle; “—” indicates antagonizing effect

We recommend that clinical researchers conduct prospective, randomized, double-blind, placebo-controlled clinical trials taking the following strategies into consideration. In our proposed scenario (Fig. 1), low dose T3 (< 15 mcg/m^2^/day) (Pingitore et al. 2019) may be initiated as adjunct therapy with currently recommended pharmacologic agents. Serum T3 levels need to be monitored to stay within the reference range. Given the relatively short half-life of T3, novel formulations (Idrees et al. 2019) of L-T3 may include slow-release tablets, liquid solutions, or soft gel capsules (modified T3; modT3) as reported (Pingitore et al. 2023; Salvatore et al. 2022; Idrees et al. 2019; Dumitrescu et al. 2022). Although such modT3 formulations are not currently available, supportive clinical trials could hasten their development. Measurements of serum NT-proBNP and T3/T4 ratio would be a valuable tool to identify responders (especially, if the sole intervention is T3) and to monitor dosing and scheduling of the treatment by testing BNP immediately before treatment and specific intervals after treatment initiation.

The use of T3 plus T4 combination therapies has increased over the past decade for primary hypothyroidism. Importantly, there were no significant CV adverse reactions (Table-1) with L-T4 + L-T3 or desiccated thyroid extract (DTE) despite the increase in serum T3 levels in some cases. Recently, a double-blind, randomized, controlled trial of L-T3 plus L-T4 treatment showed favorable changes in diastolic function over 12 months without any adverse CV events in athyreotic low-risk thyroid cancer patients (Biondi et al. 2023). In addition, a recent prospective study (Buber et al. 2023) investigating females with hypothyroidism reported improvements in left atrial volume index and atrial conduction times with the combined therapy. Furthermore, in a study (Penna et al. 2024) of 5106 adults, after covariate balancing, no significant differences were observed in healthcare utilization related to CV disorders and bone health, between L-T4-only versus combination therapies (L-T3 plus L-T4 or DTE). In an observational retrospective study (from 2010 to 2016) of 2400 patients with hypothyroidism receiving L-T4 monotherapy including a subgroup of 100 patients receiving combination therapies of L-T4 + L-T3 or DTE, no additional risks were observed for AF, CV disease, or mortality in patients of all ages (Tariq et al. 2018). A systematic review, meta-analysis, meta-regression, and network meta-analysis of eleven randomized controlled trials (de Lima Beltrao et al. 2025) from eight cross-over studies with a total of 1,135 adult patients comparing treatments for hypothyroidism (L-T4 monotherapy vs. L-T4 + L-T3 or DTE combination therapies) was recently published. Data extraction and quality assessment conducted independently by four researchers using PubMed, Embase, and Cochrane databases showed that patients significantly preferred combination therapy over T4 monotherapy. As mentioned earlier, the long-term Scotland-based observational study comparing patients consuming L-T4 only (*n* = 33,955) vs. L-T4 + L-T3 (*n* = 327) or L-T3 only (*n*= 73) with a mean follow-up of 9.3 years did not show higher mortality or morbidity risk associated with CV disease, AF, DM, or fractures in patients taking L-T3 (alone or in combination) compared to LT4 only. This indicates that L-T3 is not deleterious for CV health in the long-term (Leese et al. 2016). In a prospective, randomized, double-blind, crossover study (Shakir et al. 2021), subgroup analyses of the most symptomatic primary hypothyroidism patients on L-T4-only treatment also showed significant improvements in their symptoms, quality-of-life, depression, and memory after switching to combination therapy containing T3 plus T4.

**Table 1:** Cardiovascular effects of combination therapies of L-T4 and L-T3: L-T3 - Liothyronine; L-T4 -Levothyroxine; DTE - Desiccated Thyroid Extract; CV - Cardiovascular; BP - Blood Pressure

Study, Year, Country	Design	Number of Patients	Mean Age (years)	Comparison Groups	Major Outcomes	Follow-Up Duration (months)
Biondi B, et al.(Biondi et al. 2023), Italy	Parallel	88	44	L-T4 + L-T3, L-T4, and Euthyroid	L-T3 + L-T4 treatment improved diastolic function without adverse CV events compared to L-T4 + Placebo.	12
Buber I, et al.(Buber et al. 2023), Turkey	Interventional	47	41	L-T4 + L-T3, and L-T4	L-T3 + L-T4 treatment led to significant improvements in left atrial volume index and atrial conduction times with no change in heart rate.	6
Fadeyev VV, et al.(Fadeyev et al. 2010), Russia	Parallel	36	41	L-T4 + L-T3, and L-T4	Decreased both total and LDL cholesterol only in the L-T4 + L-T3 group and not in the L-T4-only group compared to their respective baselines	6
Appelhof BC, et al.(Appelhof et al. 2005) , The Netherlands	Parallel	140	49	L-T4 + L-T3 (10:1), L-T4 + L-T3 (5:1), and L-T4	Cholesterol was reduced in both L-T4/L-T3 groups and not in L-T4-only group. Heart rate was only minimally accelerated with L-T4/L-T3 5:1 (76 bpm) and not in L-T4/L-T3 10:1 or L-T4-only groups. Systolic BP reduced only in L-T4 group.	3.5
Leese GP, et al.(Leese et al. 2016), Scotland	Observational	34,355	53.6	L-T4 + L-T3, L-T3, and L-T4	No significant increase in mortality or morbidity risk associated with CV disease, AF, DM, or fractures	111.6
Brigante G, et al.(Brigante et al. 2024), Italy	Parallel	121	56	L-T4 + L-T3, and L-T4	L-T4 + L-T3 non-significantly reduced total cholesterol and triglycerides compared to placebo-treated L-T4 group.	5.5
Penna GC et al.(Penna et al. 2024), United States	Cross-sectional study	5437	59.4	L-T4 + L-T3, L-T4, and DTE	No significant differences in healthcare utilization related to CV disorders between L-T4-only versus combination therapies (L-T3 plus L-T4 or DTE).	~ 48
Bunevičius R, et al.(Bunevicius et al. 1999), Lithuania	Cross-over	33	46	L-T4 + L-T3, and L-T4	Decrease in systolic (6 mmHg) and diastolic (2 mmHg) blood pressures in combination therapy (*p* > 0.05) vs. L-T4 only	1.15
Escobar-Morreale HF, et al.(Escobar-Morreale et al. 2005), Spain	Cross-over	26	48	L-T4 + L-T3, and L-T4	Decrease in heart rate and cardiac output in combination therapy (*p* < 0.05) vs. L-T4 only	1.84
Shakir MKM, et al.(Shakir et al. 2021), United States	Cross-over	75	50	L-T4 + L-T3, L-T4, and DTE	Heart rate was only minimally accelerated with DTE (73.7 bpm) and not in the other groups	5.06
Tariq A, et al.(Tariq et al. 2018), United States	Observational	100	54	L-T4 + L-T3, L-T4, and DTE	No additional risks of atrial fibrillation, CV disease, or mortality in patients of all ages with hypothyroidism.	27
Walsh JP, et al.(Walsh et al. 2003), Australia	Cross-over	101	48	L-T4 + L-T3, and L-T4	No significant CV adverse effects	2.3

Consideration of TH dysfunction in the pathology of HF and considering T3/T4 therapy as standardized management of these patients may still require carefully designed clinical trials that include patients from diverse backgrounds, disease severity, age, and sex to ascertain optimal drug dosing and scheduling for specific patient subgroups. Notably, results are pending from an NIH-approved randomized, double-blind, placebo-controlled, cross-over study (NCT04111536) testing efficacy of L-T3 treatment of patients with HFpEF and LT3S.

Thyroid dysfunction underlies many chronic diseases that are projected to significantly increase in the future. Thus, to lessen the morbidity and mortality associated with these conditions and to reduce the societal burden on healthcare costs, new treatment approaches to improve patients’ quality of life with heart disease as we have discussed here, are warranted.

## Data Availability

No datasets were generated or analysed during the current study.

## Abbreviations

AF: Atrial Fibrillation
ATA: American Thyroid Association
BNP: B-type Natriuretic Peptide
CI: Cardiac Index
CV: Cardiovascular
DM: Diabetes Mellitus
DTE: Desiccated Thyroid Extract
EF: Ejection Fraction
ETA: European Thyroid Association
fT4: Free Thyroxine
HF: Heart Failure
HFpEF: Heart Failure with Preserved Ejection Fraction
Ito: transient outward potassium current
IKs: slowly activating delayed rectifier potassium current
L-T3: Liothyronine (Synthetic T3)
L-T4: Levothyroxine
LV: Left Ventricle
LT3S: Low Triiodothyronine Syndrome
MI: Myocardial Infarction
modT3: Modified T3
NICE: National Institute for Health and Care Excellence
NT-proBNP: N-terminal pro–B-type Natriuretic Peptide
RR: Relative Risk
rT3: Reverse T3
SCD: Sudden Cardiac Death
SCH: Subclinical Hypothyroidism
T3: Triiodothyronine
T4: Thyroxine; Tetraiodothyronine
TH: Thyroid Hormone
THIRST: Thyroid Hormone Replacement Therapy in ST Elevation Myocardial Infarction
ThyrAMI: Thyroxine in Acute Myocardial Infarction
TRICC: Triiodothyronine for Infants and Children Undergoing Cardiopulmonary Bypass
TSH: Thyroid-Stimulating Hormone
